# *Mycobacterium abscessus* isolated from municipal water - a potential source of human infection

**DOI:** 10.1186/1471-2334-13-241

**Published:** 2013-05-25

**Authors:** Rachel Thomson, Carla Tolson, Hanna Sidjabat, Flavia Huygens, Megan Hargreaves

**Affiliations:** 1 Gallipoli Medical Research Centre, Greenslopes Private Hospital, Brisbane, QLD, Australia; 2 QLD Mycobacterial Reference Laboratory, Pathology Queensland, RBWH Campus, Herston Rd, Herston, QLD 4006, Australia; 3 University of Queensland Centre for Clinical Research, Herston Rd, Herston, QLD 4006, Australia; 4 Institute of Health and Biomedical Innovation, Kelvin Grove Campus, Queensland University of Technology, Brisbane, QLD 4059, Australia; 5 Queensland University of Technology, Faculty of Science and Technology, George Street, Brisbane, QLD 4001, Australia

## Abstract

****Background**:**

*Mycobacterium abscessus* is a rapidly growing mycobacterium responsible for progressive pulmonary disease, soft tissue and wound infections. The incidence of disease due to *M. abscessus* has been increasing in Queensland. In a study of Brisbane drinking water, *M. abscessus* was isolated from ten different locations.

The aim of this study was to compare genotypically the *M. abscessus* isolates obtained from water to those obtained from human clinical specimens.

**Methods:**

Between 2007 and 2009, eleven isolates confirmed as *M. abscessus* were recovered from potable water, one strain was isolated from a rainwater tank and another from a swimming pool and two from domestic taps. Seventy-four clinical isolates referred during the same time period were available for comparison using *rep-*PCR strain typing (Diversilab).

**Results:**

The drinking water isolates formed two clusters with ≥97% genetic similarity (Water patterns 1 and 2). The tankwater isolate (WP4), one municipal water isolate (WP3) and the pool isolate (WP5) were distinctly different. Patient isolates formed clusters with all of the water isolates except for WP3. Further patient isolates were unrelated to the water isolates.

**Conclusion:**

The high degree of similarity between strains of *M. abscessus* from potable water and strains causing infection in humans from the same geographical area, strengthens the possibility that drinking water may be the source of infection in these patients.

## Background

Nontuberculous mycobacteria are environmental organisms that can cause progressive lung disease in susceptible patients. *M. abscessus* is a significant problem as it is highly resistant to antimicrobial agents and usually requires prolonged treatment (>six months) with intravenous and oral antibiotics in combination [1-3]. The infection is often relentless despite treatment, and even if treatment is apparently effective, relapse is common. Rapidly growing mycobacteria such as *M. abscessus* are also well documented causes of skin and soft tissue infections, especially complicating surgical procedures and injection sites [4].

Water is an important environmental reservoir of mycobacteria causing human disease [5-7]. Humans are exposed to waterborne mycobacteria through drinking, swimming and bathing. Also aerosols generated during these activities may be inhaled, [8] potentially resulting in disease. There is a recognized association between pulmonary infection with rapid growing mycobacteria [9,10] (*M. abscessus* and *M. fortuitum*) and esophageal disorders. It is possible that patients acquire infection by aspirating contaminated water [11]. Outbreak investigations have identified *M. abscessus* in hospital water, dialysis [12,13] and surgical equipment and endoscopy cleaning equipment [14,15]. In many of these instances it is not clear whether the source of these outbreaks was an infected patient or water.

The taxonomy of *Mycobacterium abscessus* has been a topic of debate in the literature. *M. massiliense* and *M. bolletii* cannot be separated from *M. abscessus* on the basis of extensive phenotypic analysis and genotypic studies have lead to the proposition that the three taxa represent a single species with internal variability [16]. Strain variation amongst species of mycobacteria is well known and Pulsed Field Gel Electrophoresis (PFGE) has been considered the “Gold standard” for strain typing of many mycobacteria. ERIC (enterobacterial repetitive intergenic consensus) PCR has been used successfully to differentiate strains of mycobacteria associated with outbreaks of disease in mesotherapy clinics due to *M. abscessus* and *M. chelonae*, and in post mammoplasty patients with *M. fortuitum* infections [17,18]. A high concordance with PFGE was shown. More recently *rep*-PCR (Diversilab) was used in comparison to PFGE in a suspected outbreak of *M. abscessus* in a cystic fibrosis clinic [19]. Isolates that were identical on PFGE shared only 90% similarity with rep-PCR, suggesting that the latter method may be more discriminatory. *Rep*-PCR was similarly shown to concur with strain typing using VNTR (variable number tandem repeats) [20]. This method has significant advantages in cost and time saving over other methods and has demonstrated high discriminatory power.

In Queensland NTM disease remains notifiable and a central reference laboratory performs speciation of all culture positive isolates, providing a unique opportunity to capture isolates from around the state. The incidence of disease due to NTM has been increasing [21] and between 2007–2008 water sampling was conducted to investigate the presence of pathogenic NTM in potable water [22]. This paper reports the comparison of these water isolates with human isolates collected from patients in South east Queensland during the same time period.

## Methods

Water isolates obtained in previous studies [22] had been stored in Dubos broth at −20°C and were thawed and subbed onto 7H11 plates as well as Lowenstein-Jensen slopes and incubated at 35°C until sufficient growth was available.

Those identified using 16S rDNA sequencing as *M. abscessus/M. chelonae* underwent *hsp*65 and *rpo*B gene fragment sequencing for more definitive identification. According to the proposed changes by Leao et al. [16] in line with the Bacteriological code, for the purposes of this study all isolates identified as *M. abscessus, M. bolletii, M. massiliense* were included as *M. abscessus* species.

Human samples that were collected as part of routine patient care, were digested and decontaminated using 4% NaOH, neutralized with phosphoric acid and centrifuged at 3000g to concentrate the acid-fast bacilli (AFB). Smears were prepared from the sediment and stained by the Ziehl-Niehlsen (ZN) method. One Lowenstein-Jensen slope (±pyruvate) and 7ml Mycobacterial Growth Indicator Tube (MGIT) were inoculated and incubated at 35°C until growth was detected. ZN staining of colonies confirmed AFB. Multiplex PCR was performed to discriminate between *M. tuberculosis, M. avium, M. intracellulare, M. abscessus* and other *Mycobacterium spp.* Isolates identified as other *Mycobacterium spp* were further speciated using Hain Life Sciences GenoType® Mycobacterium CM kit (2004–7 only) and/or 16S rDNA sequencing in conjunction with phenotypic characteristics. All clinical isolates underwent gene fragment sequencing for *hsp*65 and *rpo*B.

### Strain typing using automated Rep-PCR

The clonality of clinical and water *M. abscessus* isolates and a control strain (ATCC 19977) was determined using a *rep*-PCR based method (Diversilab® system, bioMerieux, Melbourne). DNA was extracted from clinical and water isolates using the Ultraclean Microbial DNA Isolation Kit (Mo Bio Laboratories, CA, USA). PCR mixture was prepared using AmpliTaq polymerase and PCR buffer (Applied Biosystems, New Jersey, USA) and Mycobacterium Diversilab® primer mix according to the manufacturer’s instructions. Separation and detection of *Rep*-PCR products was performed by micro-fluidic chips of the Diversilab® System. A laboratory control strain of *M. abscessus* was included, and two chips were repeated because of low intensity banding. In most cases when the intensity improved the pattern didn’t change from that obtained with low intensity. An additional chip was run twice to confirm reproducibility of strain patterns. Any aberrances were excluded from the analysis. Fingerprints were analysed with Diversilab® software v.3.4.38 using the Pearson correlation co-efficient and unweighted pair group method with arithmetic means to compare isolates and determine clonal relationship. A similarity index of ≥97% was used to define isolates that were indistinguishable, based on the manufacturers recommendations despite previously published comparisons of *rep*-PCR with both PFGE and Variable Number Tandem Repeats, [20] where a cut off of 90% was used. Isolates that shared 95% similarity were considered similar (and formed groups), and those <90% considered ‘different/unrelated’.

The study protocol was approved by the Human Research Ethics Committee of The Prince Charles Hospital (EC-2617).

## Results

During the 10 years from 2001–2010, there were 486 patient notifications: 68.1% were pulmonary, 22.2% cutaneous or soft tissue (Table 1).

**Table 1:** **Sites of isolation of *M. abscessus *from human samples 2001-2010**

**Site of isolate**	**Frequency (%)**
Blood	14 (2.9)
Bones and joints	5 (1)
Cutaneous/Soft tissue	108 (22.2)
Eye	1 (0.2)
Lymph node + Other	1 (0.2)
Lymph nodes	3 (0.6)
Peritoneal	2 (0.4)
Post surgical/Medical access device	19 (3.9)
Pulmonary	331 (68.1)
Unknown	2 (0.4)
Total	486 (100)

Seventy-four clinical isolates collected during 2007–8 were available for comparison with fifteen water isolates. The mean age of patients was 55.94 yrs (SD 22.25; median 58.5; range 2-90yrs). Fifty percent were male. Forty-four patients had clinically significant pulmonary disease (according to clinician notification and the ATS/IDSA criteria), 11 patients had pulmonary isolates that were considered contaminants or not clinically significant and 19 patients had isolates from extra-pulmonary sites.

Eleven patients (14.9%) were included from other parts of Australia who lived >500 km outside the Brisbane water distribution network, and would have received potable water supply to their homes from different networks. Of the remaining patients, 31/63 (49.2%) patients lived in reservoir zones that contained sample points that grew *M. abscessus*.

Of 19 water isolates identified as *M. abscessus/M. chelonae* using 16s rDNA sequencing, 17 underwent successful *hsp*65 and *rpo*B sequencing. This identified 14 isolates as *M. abscessus* and one as *M. bolletii –* all considered *M. abscessus subs abscessus* according to the proposed taxonomy by Le*ao*[16]. There was insufficient DNA in two samples. Fifteen water isolates of *M. abscessus* were strain typed using *rep*-PCR. Eleven isolates came from six different potable water distribution system sampling sites; two came from domestic taps in the same dwelling, one came from an unrelated rainwater tank and the other from a suburban domestic swimming pool.

**Figure 1:**
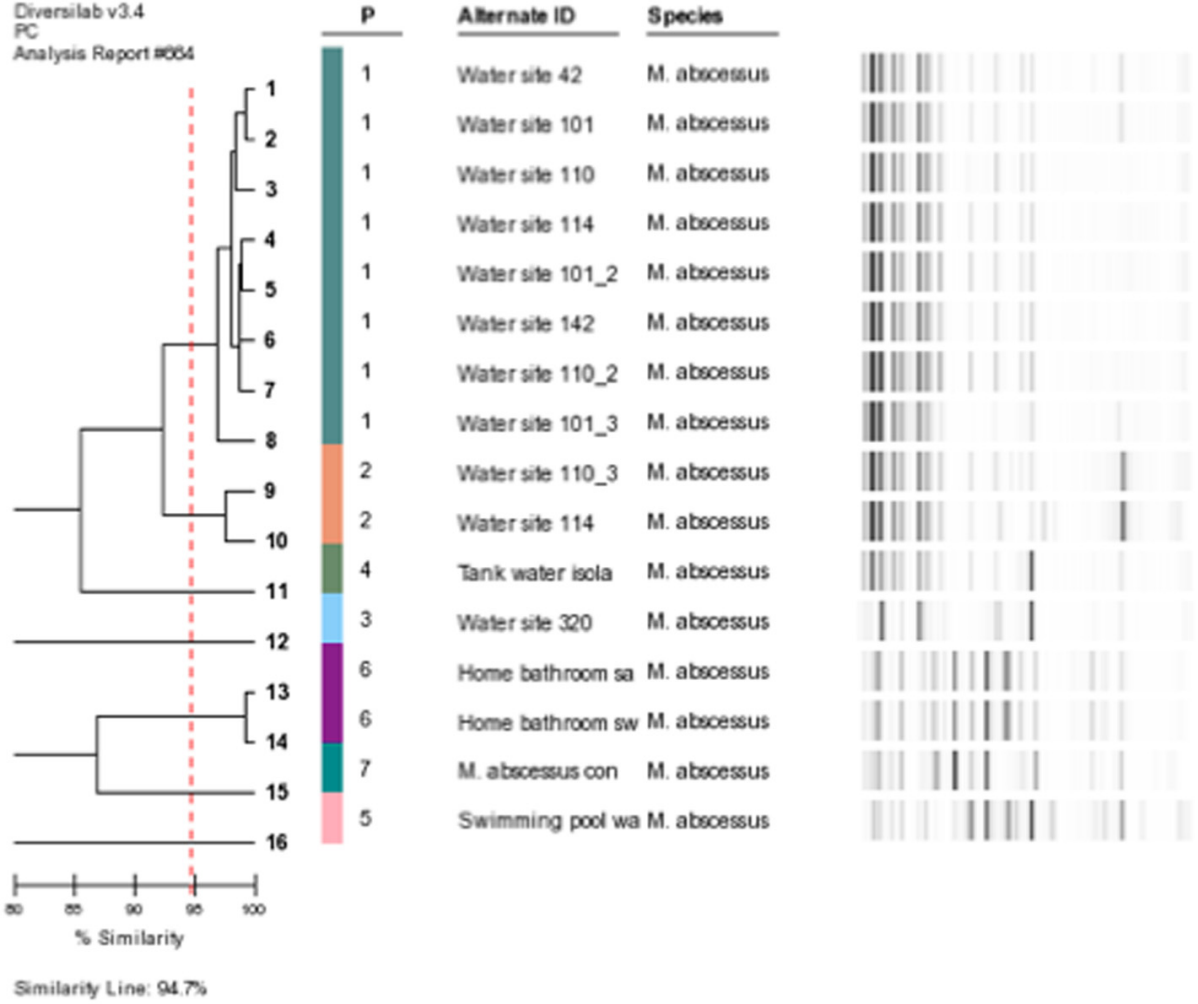
***rep*-PCR dendrogram of *M. abscessus *water strains with Pearson Correlation Analysis.**

The 15 water isolates formed 6 different patterns (Figure 1). Municipal water isolates formed two clusters of indistinguishable isolates (≥97% similarity) and were classified as Group WP1 (eight isolates) and Group WP2 (two isolates) and a single isolate WP3. The rainwater tank isolate (Group WP4) and the swimming pool isolate (Group WP5) were distinctly different. The two isolates from a patient’s domestic taps were indistinguishable (WP 6) (Figure 1).

When the clinical isolates were analysed with the water isolates there were 28 patterns/clusters that shared >97% similarity (indistinguishable). Thirteen of these clusters contained more than 1 isolate, and the remaining patterns were single isolates only. The indistinguishable clusters that were similar (>95% similarity) were then grouped into seven main groups. Full report and graphics in Additional file 1.

**Figure 2:**
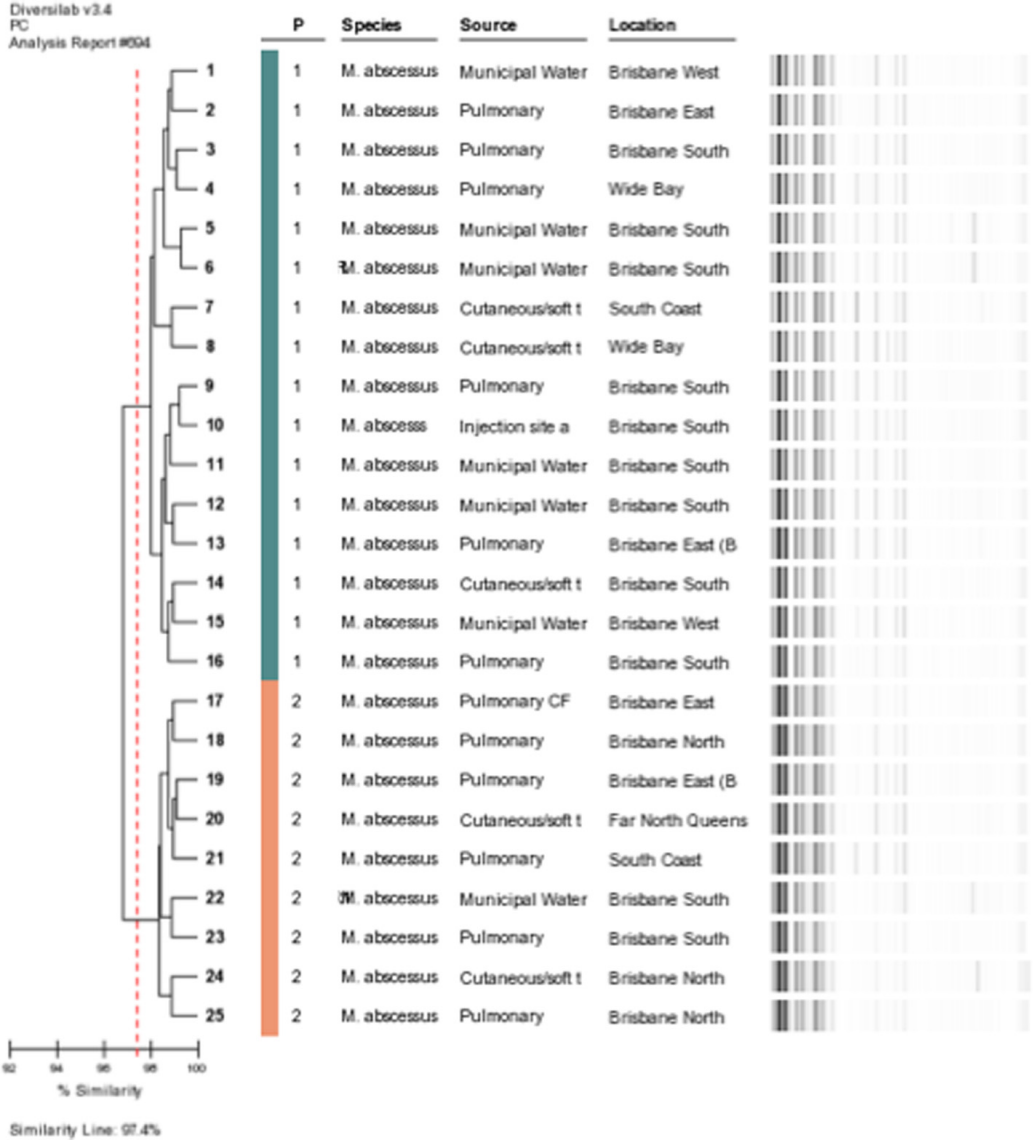
***rep*-PCR dendrogram of examples of main clinical strain patterns 17 (1, green) and 15 (2, orange) demonstrating similarities between water and clinical isolates.** (Full dendrogram in Additional file 1).

There were two large similar clusters of 21 (P15) and 22 (P17) isolates respectively. Examples demonstrated in Figure 2. P15 included one of the municipal water isolates from WP1 and P17 contained the remaining seven isolates from WP1. A further three clusters (P12-14) containing 5 clinical isolates were similar to cluster P15 and a single isolate P16 was similar to P17.

**Figure 3:**
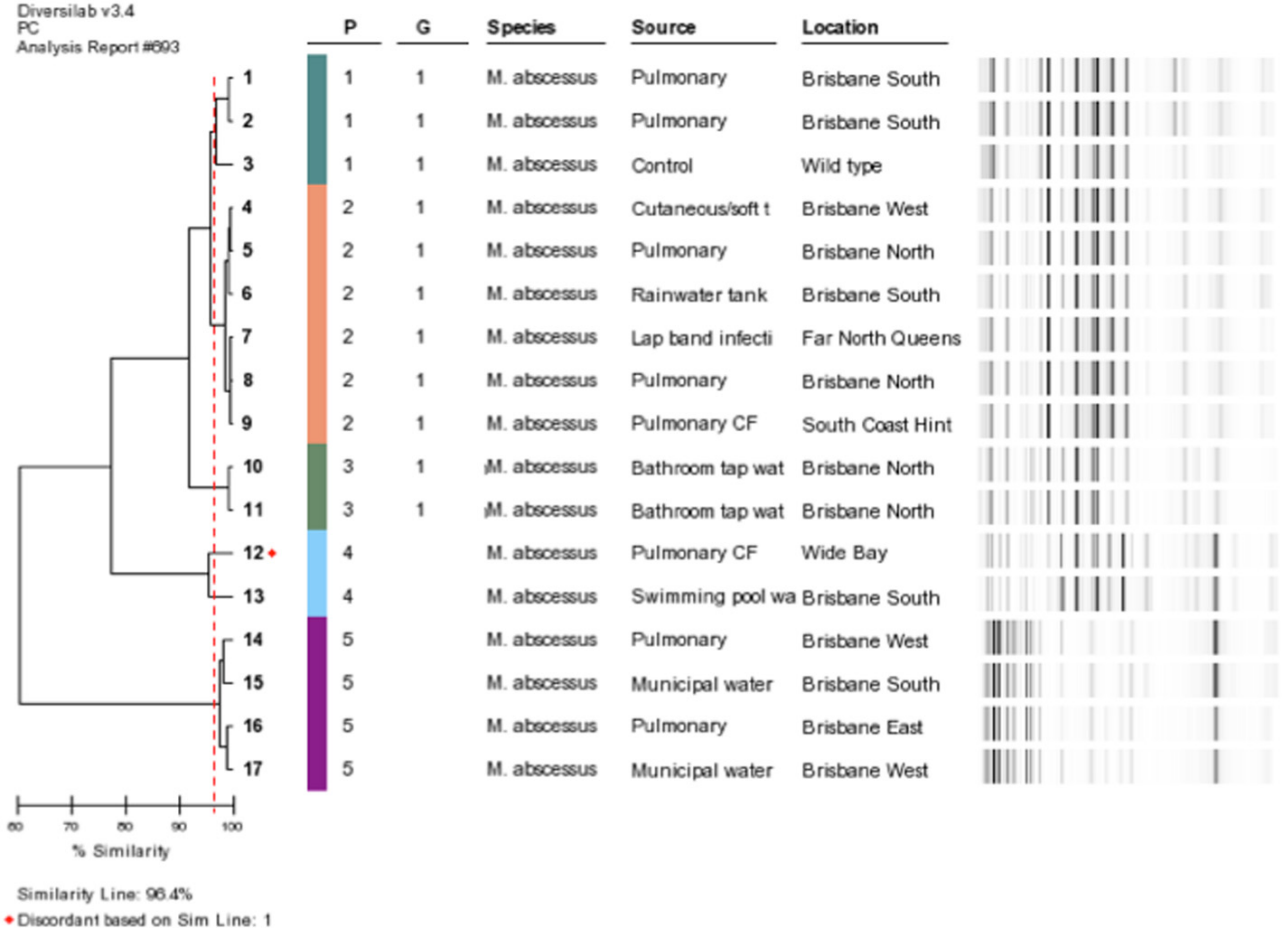
***rep*-PCR dendrogram showing examples of different strain patterns where there were similarities between clinical and water strains.**

The two water isolates from WP2 also clustered with six clinical isolates (P20-22;Gp 4: Additional file 1). Five clinical isolates were indistinguishable from the tank water isolate (P2). A further three clinical isolates and the two domestic tap water isolates were similar to this cluster (Group 1). One clinical isolate was indistinguishable from the swimming pool isolate Figure 3.

The isolate from water site 320 (WP3) differed from all the clinical isolates.

There were no significant differences between the characteristics of groups of patients in each strain cluster (age, gender, type of disease, site of infection, ground water source or reservoir zone) (Table 2). Some of the isolates from more remote areas did have unique strain patterns, but equally so did some of the Brisbane area isolates. Some of the remote area isolates were similar to those of patients from Brisbane. However as is often the case with NTM infections, the precise location of where infection is acquired can be difficult to pinpoint.

**Table 2:** **Clinical details of patients with *M. abscessus *isolates used for strain typing comparison**

	**n (%)**	Age* Mean±SD	**Gender M:F**	** Species**
Lung	54 (73)	58.24±24.53	27:27	*M. abscessus subs abscessus* 34 (63%)
				*M. abscessus subs bolletii* 20 (37%)
Cutaneous/Soft Tissue Infection	11 (14.9)	49.18±14.49	5:6	*M. abscessus subs abscessus* 9 (81.8%)
				*M. abscessus subs bolletii* 2 (18.2%)
Invasive Device/Line	3 (4.1)	42.67±19.63	2:1	*M. abscessus subs abscessus* 3 (100%)
Surgical Wound Infection	2 (2.7)	46.00±7.07	1:1	*M. abscessus subs abscessus* 1 (50%)
				*M. abscessus subs bolletii* 1 (50%)
Bloodstream Infection (Line Assoc)	1 (1.4)	47.00±19.86	1:0	*M. abscessus subs abscessus* 1 (100%)
Injection Site	3 (4.1)	41.33±22.75	2:1	*M. abscessus subs abscessus* 3 (100%)
**Total**	**74 (100)**	**55.09±22.88**	**38:36**	***M. abscessus subs abscessus *51 (68.9%)**
				***M. abscessus subs bolletii *23 (31.1%)**

## Discussion

*M. abscessus* has become an increasingly important clinical problem in the last 10 years, and its presence in potable water has not previously been emphasized.

In the majority of published studies looking for NTM in water, no *M. abscessus* was documented [23]. However there have been taxonomical changes that have led to *M. abscessus* being recognized as independent from *M. chelonae*, so older studies reporting *M*. *chelonae* may not necessarily have differentiated *M. chelonae subsp abscessus*, particularly as both species have identical 16s rDNA sequences. But in studies of potable water done since 2000 *M. abscessus* has been rarely reported.

A study done in Pretoria, South Africa in 2004 [24] reported an analysis of 78 biofilm and water samples – 14 had NTM – no MAC, one had *M. abscessus* (16s rDNA sequencing only). In a multi-hospital outbreak investigation in Taiwan, *M. abscessus* was grown from one water sample from one of the hospitals involved however this differed on strain typing from the isolates found in patients [25]. Falkinham [26] in 2001 sampled 8 different water treatment systems (raw, treated and Distribution System (DS) samples). *M. abscessus* was only found in one raw water sample from one system. There were no rapid growers in treated water, two rapid growers were found in the DS samples but the paper doesn’t clarify the species. In a more recent study (2009), van Ingen reported no *M. abscessus* in shower or tap water in the Netherlands [27]. We have found that the inclusion of liquid media increased the yield for *M. abscessus* from potable water samples [22]. However three of the 13 isolates were only grown on solid media – suggesting that for future studies using culture-based techniques, both media forms should be included.

It has long been recognized that outbreaks of disease due to rapid growers may have originated in hospital and tap water, particularly for *M. chelonae* and *M. fortuitum*[28] though in some studies the authors have acknowledged that speciation separating *M. abscessus* has not occurred. Outbreak investigations have linked *M. abscessus* infections and pseudo infections to hospital tap water, and endoscope cleaning fluids [29,30].

In all of these hospital outbreaks, the potential for contamination of hospital sources and equipment by infected patients or other environmental sources (such as dust or dirt) exists; hence the origin of the culprit strain remains unclear.

Zelazny [31] compared partial sequencing of *rpo*B, *hsp*65 and *sec*A gene fragments and *rep-*PCR (Diversilab) in order to differentiate *M. abscessus* from subspecies formerly named *M. massiliense* and *M. bolletii*. They were able to group the majority of *M. abscessus* complex clinical isolates into a group similar to the ATCC strain 19977, with a degree of diversity as low as 80% similarity to the reference strain. There were two other strain groups neither of which resembled the strain patterns obtained in our study. The geographic origin of the patients included in this study was not reported nor examined, but as the study was performed by a tertiary referral centre many came from patients around the United States (A. Zelazny, personal communication). In our study the strains obtained both from patients and from water were closely related, and differed from the strain patterns reported in the US, suggesting geographical differences in strain variation. The diversity of the US strains, may be due to the diverse origins of the isolates reported, reflecting differences in the dominant environmental strains. There was a degree of geographic diversity of our strains with unique strain types identified from central and far north Queensland (>500km and > 1000km from Brisbane respectively) and two unique strains from the bayside of Brisbane (> 30km from the CBD).

Whilst *rep*-PCR using the Diversilab has only recently been used for strain typing mycobacteria, published studies support its performance as equivalent or perhaps even more discriminatory than PFGE. Aitken et al [19] reported an outbreak of *M. abscessus* infections in a lung transplantation and cystic fibrosis center. Isolates involved were strain typed using both PFGE and *rep*-PCR (Diversilab). The isolate of the index case and outbreak cases were reported to be “genetically identical” using PFGE. The *rep*-PCR patterns of the same isolates showed only 90% genetic similarity, suggesting in fact that these strains are different, and that *rep*-PCR may be more discriminatory than PFGE.

Harris [20] describes a novel VNTR (variable number tandem repeats) method to type 41 *M. abscessus* complex strains from 17 pediatric cystic fibrosis patients and compares this with *rep*-PCR (Diversilab). As with the Aitken paper, isolates with 90% similarity were regarded as indistinguishable. The finding of six distinct profiles among 12 patients with *M. abscessus* complex suggests that the typing methods have sufficient discriminatory power.

## Conclusion

We have analysed both patient and water isolates from quite diverse and epidemiologically unrelated settings across a geographical area of >1367km^2^ and found 28 clusters. We have more definitively identified *M. abscessus* strains in potable water, as strains that are indistinguishable from those found in samples from patients known to have disease. Whilst point source investigations of outbreaks have linked *M. abscessus* infection and hospital water and equipment, the present study tightens the link between potable water, to which patients are regularly exposed, and the acquisition of infection. This has important implications for those patients at risk. Given the difficulty in treating these infections and the devastating effects they have for patients, efforts to identify effective disinfection methods for *M. abscessus* should be a high priority.

## Competing interests

All authors declare that they have no competing interests.

## Authors’ contributions

RT conceived and designed the study, processed the water samples, performed the Diversilab analysis and wrote the manuscript. CT processed water and human samples, performed DNA extraction and *rep*-PCR. HS performed *rep-*PCR experiments with CT. FH and MH contributed to the design and conduct of the study, the analysis of the results, and the writing of the manuscript. All authors reviewed and approved the final manuscript.

## Pre-publication history

The pre-publication history for this paper can be accessed here:

http://www.biomedcentral.com/1471-2334/13/241/prepub

## Supplementary Material

Additional file 1 Full Diversilab rep-PCR analysis of water and human isolates. Click here for file
