# Supplementary material for: Mycobacterium abscessus isolated from municipal water - a potential source of human infection
Source: BMC Infect Dis. 2013 May 25;13:241. doi: 10.1186/1471-2334-13-241 (PMC3668184; doi:10.1186/1471-2334-13-241)
Supplement: Additional file 1 — Full Diversilab rep-PCR analysis of water and human isolates. [file 1471-2334-13-241-S1.pdf]

UQCCR + PATH QLD RBH

Name: Report #696: MAB Add mat 1
Date: 03/16/2013
User: Hanna Sidjabat
Type: Typing
Kit: Mycobacterium
Library: None

Group	Pattern	Key	Sample ID	Species	Source	Location	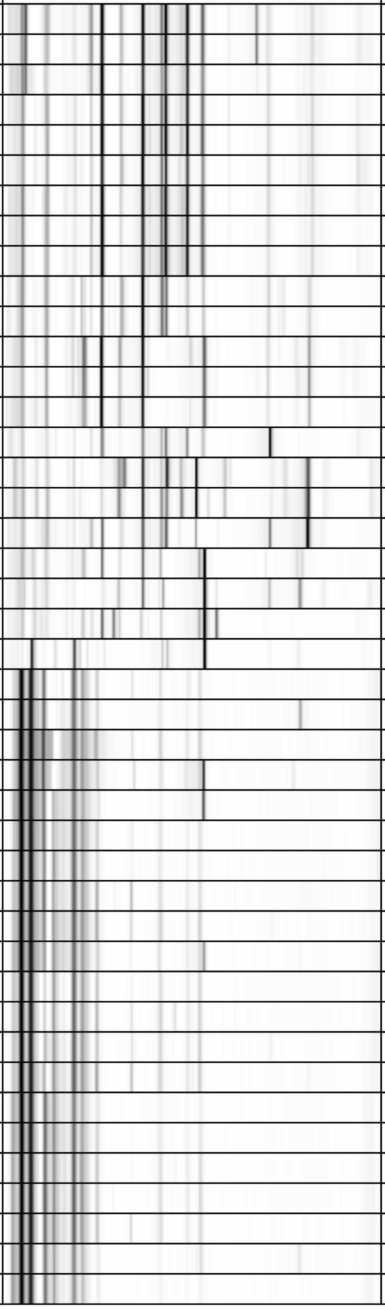	Note
1	1	1	483134518R	M. abscessus	Pulmonary	Brisbane Sou		
		2	483132823R	M. abscessus	Pulmonary	Brisbane Sou		
		3	29457824R	M. abscessus	Control	Wild type		
	2	4	450617067R	M. abscessus	Pulmonary	Brisbane Nor		
		5	379715752R	M. abscessus	Cutaneous/so	Brisbane Wes		
		6	Tank BWCPCR	M. abscessus	Rainwater ta	Brisbane Sou		
		7	483138005R	M. abscessus	Pulmonary	Brisbane Nor		
		8	274808657R	M. abscessus	Lap band inf	Far North Qu		
		9	454458408R	M. abscessus	Pulmonary CF	South Coast		
	3	10	Myco_BH Bath	M. abscessus	Bathroom tap	Brisbane Nor		
		11	Myco_BH Bath	M. abscessus	Bathroom tap	Brisbane Nor		
	4	12	360024143R	M. abscessus	Pulmonary	Brisbane Eas		
		13	419312442R	M. abscessus	Pulmonary	South Coast		
		14	M.abscessus	M. abscessus	Laboratory	Control		
	5	15	379714981R	M. abscessus	Pulmonary	Brisbane Nor		
	6	16	MCG Pool Wat	M. abscessus	Swimming poo	Brisbane Sou		
		17	492604092	M. abscessus	Pulmonary CF	Wide Bay		
	7	18	324036586	M. abscessus	Pulmonary	Brisbane Sou		
	8	19	483127533R	M. abscessus	Pulmonary	Central QLD		
	9	20	505126835	M. abscessus	Pulmonary CF	Brisbane Eas		
	10	21	505090218	M. abscessus	Pulmonary CF	Brisbane Eas		
	11	22	Sp 320_CPC 1	M. abscessus	Municipal Wa	Brisbane Wes		
2	12	23	255815033	M. abscessus	Pulmonary	Brisbane Nor		
		24	318566653	M. abscessus	Bloodstream	Brisbane Cen		
	13	25	275134670	M. abscessus	Pulmonary	Brisbane Nor		
	14	26	393818813	M. abscessus	Pulmonary	Brisbane Nor		
		27	275134456	M. abscessus	Device/Line	Central QLD		
	15	28	328446647	M. abscessus	Pulmonary	Wide Bay		
		29	328445363	M. abscessus	Pulmonary	Brisbane Nor		
		30	417748364rep	M. abscessus	Pulmonary	Brisbane Sou		
		31	29386674	M. abscessus	Soft Tissue	Brisbane Sou		
		32	291938261	M. abscessus	Pulmonary	Brisbane Nor		
		33	275135839	M. abscessus	Pulmonary	Brisbane Cen		
		34	321221479	M. abscessus	Pulmonary	Brisbane Nor		
		35	318543716	M. abscessus	Pulmonary	West Moreton		
		36	404271453	M. abscessus	Pulmonary	Brisbane Sou		
		37	328449605	M. abscessus	Pulmonary CF	Brisbane Eas		
		38	328447721	M. abscessus	Pulmonary	Brisbane Nor		
		39	275136432rep	M. abscessus	Pulmonary	Brisbane Eas		
		40	275135807rep	M. abscessus	Cutaneous/so	Far North Qu		
		41	394473438rep	M. abscessus	Pulmonary	South Coast		
		42	SP 101 WCPC_	M. abscessus	Municipal Wa	Brisbane Sou		
		43	275133084	M. abscessus	Pulmonary	Brisbane Sou		

		44	377711337	M. abscessus	Cutaneous/so	Brisbane Nor		
		45	351754090	M. abscessus	Pulmonary	Brisbane Nor		
		46	321981832	M. abscessus	Injection Si	Brisbane Eas		
		47	328446068	M. abscessus	Injection Si	Brisbane Nor		
		48	379755630rep	M. abscessus	Pulmonary CF	West Moreton		
3	16	49	409899621rep	M. abscessus	Pulmonary	Brisbane Wes		
	17	50	381048028rep	M. abscessus	Cutaneous/so	Brisbane Sou		
		51	SP142 WCPC R	M. abscessus	Municipal Wa	Brisbane Wes		
		52	328448789	M. abscessus	Pulmonary	Brisbane Sou		
		53	328440882rep	M. abscessus	Pulmonary	Brisbane Nor		
		54	271995461	M. abscessus	Pulmonary	South Coast		
		55	29389421rep	M. abscesss	Injection Si	Brisbane Sou		
		56	275139959	M. abscessus	Pulmonary	Brisbane Sou		
		57	SP114 R	M. abscessus	Municipal Wa	Brisbane Sou		
		58	29385807rep	M. abscessus	Pulmonary	Brisbane Sou		
		59	SP101 R	M. abscessus	Municipal Wa	Brisbane Sou		
		60	328558972rep	M. abscessus	Pulmonary	Brisbane Eas		
		61	SP1100207200	M. abscessus	Municipal Wa	Brisbane Wes		
		62	SP110 white	M. abscessus	Municipal Wa	Brisbane Wes		
		63	275137963	M. abscessus	Pulmonary	Brisbane Eas		
		64	311911744rep	M. abscessus	Pulmonary	Wide Bay		
		65	296555363	M. abscessus	Pulmonary	Brisbane Sou		
		66	SP101 OWPWC	M. abscessus	Municipal Wa	Brisbane Sou		
		67	SP42 WCPC R	M. abscessus	Municipal Wa	Brisbane Sou		
		68	275134359rep	M. abscessus	Cutaneous/so	Wide Bay		
		69	341211044rep	M. abscessus	Cutaneous/so	South Coast		
		70	328444147rep	M. abscessus	Surgical wou	Brisbane Sou		
		71	336395997rep	M. abscessus	Pulmonary CF	Brisbane Eas		
	18	72	318540128	M. abscessus	Pulmonary	Brisbane Nor		
	19	73	328447600rep	M. abscessus	Cutaneous/So	Brisbane Nor		
4	20	74	387640523rep	M.a bscessus	Surgical wou	Brisbane Cen		
		75	297683281	M. abscessus	Pulmonary	Brisbane Wes		
		76	SP110 R	M. abscessus	Municipal wa	Brisbane Wes		
		77	366921511	M. abscessus	Pulmonary	Brisbane Eas		
	21	78	379714112rep	M. abscessus	Pulmonary	Sunshine Coa		
	22	79	SP114 WCPC R	M. abscessus	Municipal wa	Brisbane Sou		
		80	280885764rep	M. abscessus	Pulmonary	Brisbane Wes		
	23	81	275138510	M. abscessus	Cutaneous/So	South Coast		
5	24	82	274804151	M. abscessus	Surgical wou	Brisbane Cen		
	25	83	274802998	M. abscessus	Cutaneous/So	South Coast		
6	26	84	275136552	M. abscessus	Pulmonary	Brisbane Nor		
		85	29387102	M. abscessus	Pulmonary	Brisbane Wes		
		86	275133697rep	M. abscessus	Pulmonary	Brisbane Sou		
	27	87	328447776rep	M. abscessus	Pulmonary	Brisbane Sou		
	28	88	390900825rep	M. abscessus	Pulmonary	Brisbane Sou		
		89	328447798rep	M. abscessus	Pulmonary	Brisbane Sou		
		90	427760051rep	M. abscessus	Pulmonary	Brisbane Nor		
		91	275134478	M. abscessus	Pulmonary	Brisbane Nor		

Reviewed by Date

Approved by	Date

Diversilab v3.4
PC
Analysis Report #696

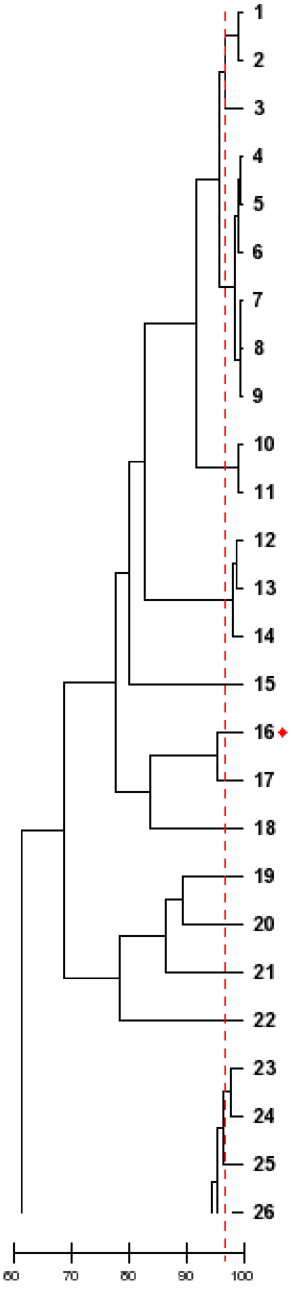

	P	G	Sample ID	Species	Source	Location
1	1	1	483134518R	M. abscessus	Pulmonary	Brisbane South
2	1	1	483132823R	M. abscessus	Pulmonary	Brisbane South
3	1	1	29457824R	M. abscessus	Control	Wild type
4	2	1	450617067R	M. abscessus	Pulmonary	Brisbane North
5	2	1	379715752R	M. abscessus	Cutaneous/soft t	Brisbane West
6	2	1	Tank BWCPCR	M. abscessus	Rainwater tank	Brisbane South
7	2	1	483138005R	M. abscessus	Pulmonary	Brisbane North
8	2	1	274808657R	M. abscessus	Lap band infecti	Far North Queens
9	2	1	454458408R	M. abscessus	Pulmonary CF	South Coast Hint
10	3	1	Myco_BH Bathroom	M. abscessus	Bathroom tap wat	Brisbane North
11	3	1	Myco_BH Bathroom	M. abscessus	Bathroom tap wat	Brisbane North
12	4		360024143R	M. abscessus	Pulmonary	Brisbane East (B
13	4		419312442R	M. abscessus	Pulmonary	South Coast
14	4		M.abscessus cont	M. abscessus	Laboratory	Control
15	5		379714981R	M. abscessus	Pulmonary	Brisbane North
16 ♦	6		MCG Pool Water R	M. abscessus	Swimming pool wa	Brisbane South
17	6		492604092	M. abscessus	Pulmonary CF	Wide Bay
18	7		324036586	M. abscessus	Pulmonary	Brisbane South
19	8		483127533R	M. abscessus	Pulmonary	Central QLD
20	9		505126835	M. abscessus	Pulmonary CF	Brisbane East (B
21	10		505090218	M. abscessus	Pulmonary CF	Brisbane East (B
22	11		Sp 320_CPC 14_6_	M. abscessus	Municipal Water	Brisbane West
23	12	2	255815033	M. abscessus	Pulmonary	Brisbane North
24	12	2	318566653	M. abscessus	Bloodstream	Brisbane Central
25	13	2	275134670	M. abscessus	Pulmonary	Brisbane North
26	14	2	393818813	M. abscessus	Pulmonary	Brisbane North

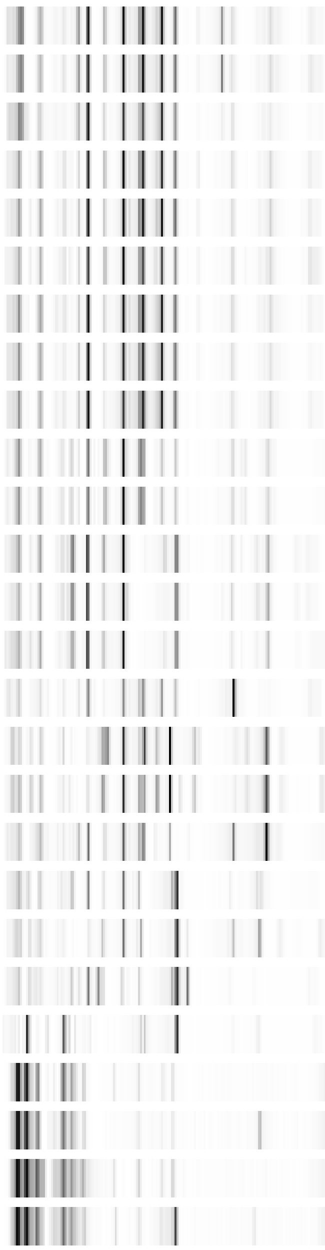

Similarity Line: 96.7%
♦ Discordant based on Sim Line: 2

Diversilab v3.4
PC
Analysis Report #696

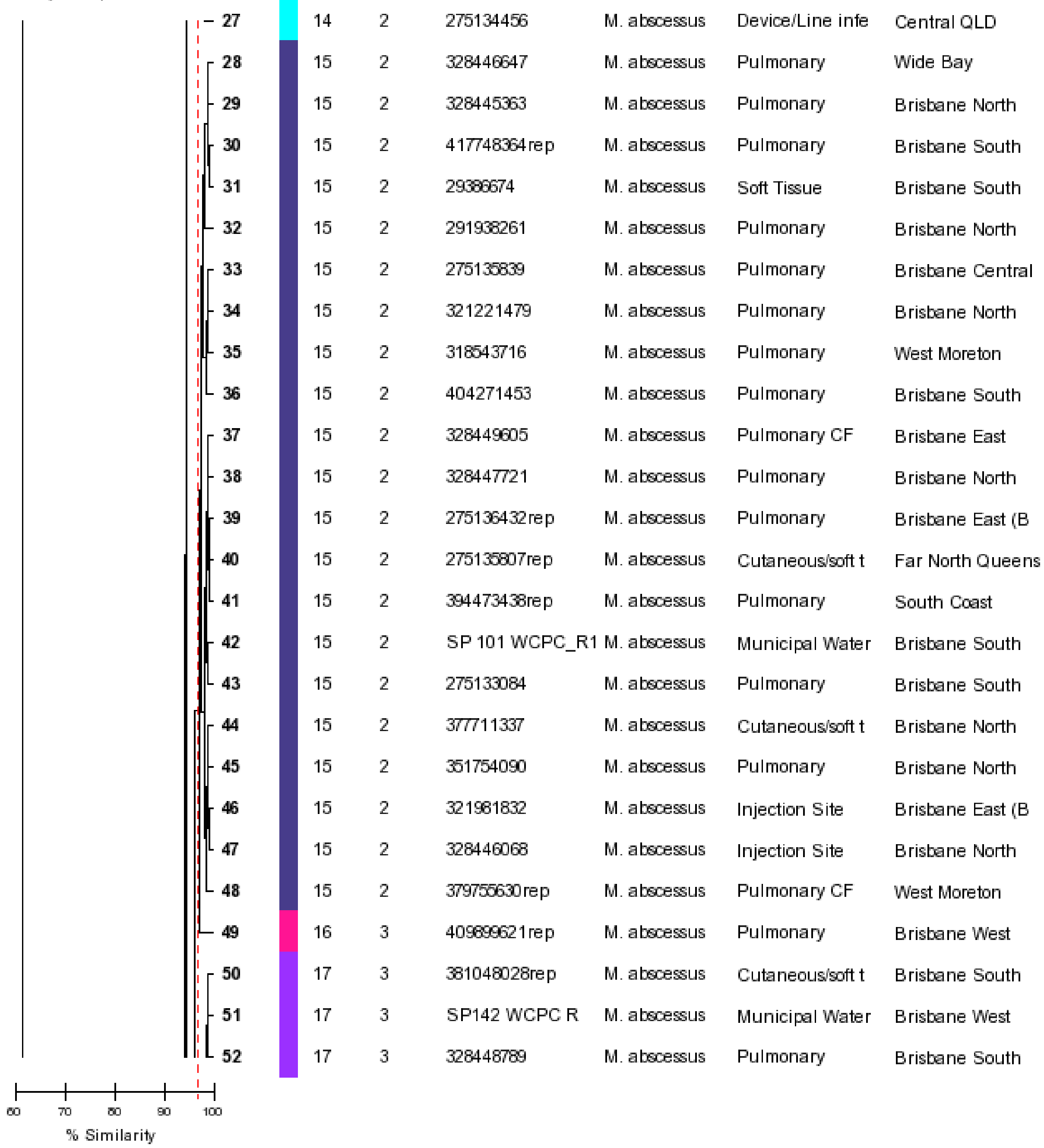

	P	G	Sample ID	Species	Source	Location
27	14	2	275134456	M. abscessus	Device/Line infe	Central QLD
28	15	2	328446647	M. abscessus	Pulmonary	Wide Bay
29	15	2	328445363	M. abscessus	Pulmonary	Brisbane North
30	15	2	417748364rep	M. abscessus	Pulmonary	Brisbane South
31	15	2	29386674	M. abscessus	Soft Tissue	Brisbane South
32	15	2	291938261	M. abscessus	Pulmonary	Brisbane North
33	15	2	275135839	M. abscessus	Pulmonary	Brisbane Central
34	15	2	321221479	M. abscessus	Pulmonary	Brisbane North
35	15	2	318543716	M. abscessus	Pulmonary	West Moreton
36	15	2	404271453	M. abscessus	Pulmonary	Brisbane South
37	15	2	328449605	M. abscessus	Pulmonary CF	Brisbane East
38	15	2	328447721	M. abscessus	Pulmonary	Brisbane North
39	15	2	275136432rep	M. abscessus	Pulmonary	Brisbane East (B
40	15	2	275135807rep	M. abscessus	Cutaneous/soft t	Far North Queens
41	15	2	394473438rep	M. abscessus	Pulmonary	South Coast
42	15	2	SP 101 WCPC_R1	M. abscessus	Municipal Water	Brisbane South
43	15	2	275133084	M. abscessus	Pulmonary	Brisbane South
44	15	2	377711337	M. abscessus	Cutaneous/soft t	Brisbane North
45	15	2	351754090	M. abscessus	Pulmonary	Brisbane North
46	15	2	321981832	M. abscessus	Injection Site	Brisbane East (B
47	15	2	328446068	M. abscessus	Injection Site	Brisbane North
48	15	2	379755630rep	M. abscessus	Pulmonary CF	West Moreton
49	16	3	409899621rep	M. abscessus	Pulmonary	Brisbane West
50	17	3	381048028rep	M. abscessus	Cutaneous/soft t	Brisbane South
51	17	3	SP142 WCPC R	M. abscessus	Municipal Water	Brisbane West
52	17	3	328448789	M. abscessus	Pulmonary	Brisbane South

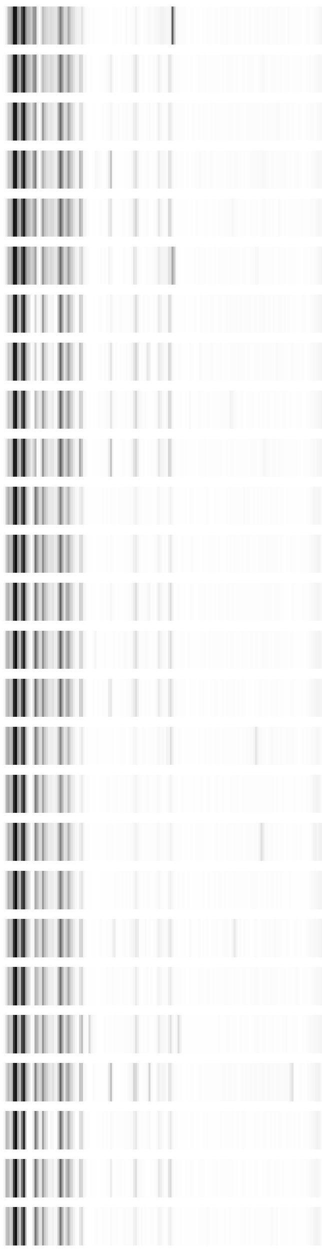

Similarity Line: 96.7%
◆ Discordant based on Sim Line: 2

Diversilab v3.4
PC
Analysis Report #696

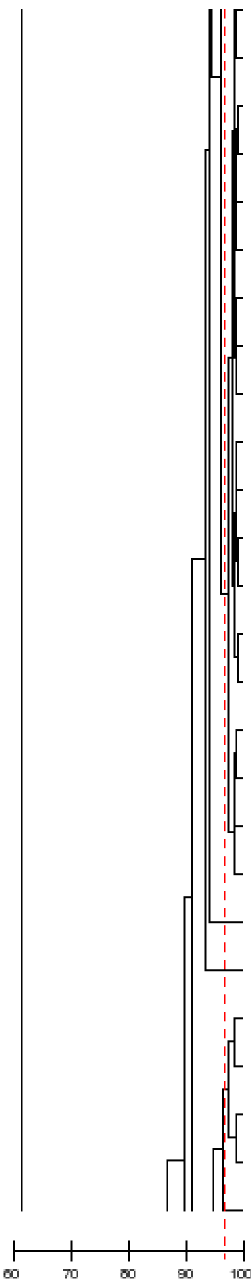

#	P	G	Sample ID	Species	Source	Location
53	17	3	328440882rep	M. abscessus	Pulmonary	Brisbane North
54	17	3	271995461	M. abscessus	Pulmonary	South Coast
55	17	3	29389421rep	M. abscesss	Injection Site	Brisbane South
56	17	3	275139959	M. abscessus	Pulmonary	Brisbane South
57	17	3	SP114 R	M. abscessus	Municipal Water	Brisbane South
58	17	3	29385807rep	M. abscessus	Pulmonary	Brisbane South
59	17	3	SP101 R	M. abscessus	Municipal Water	Brisbane South
60	17	3	328558972rep	M. abscessus	Pulmonary	Brisbane East (B
61	17	3	SP11002072007R	M. abscessus	Municipal Water	Brisbane West
62	17	3	SP110 white R	M. abscessus	Municipal Water	Brisbane West
63	17	3	275137963	M. abscessus	Pulmonary	Brisbane East
64	17	3	311911744rep	M. abscessus	Pulmonary	Wide Bay
65	17	3	296555363	M. abscessus	Pulmonary	Brisbane South
66	17	3	SP101 OWPWC R	M. abscessus	Municipal Water	Brisbane South
67	17	3	SP42 WCPC R	M. abscessus	Municipal Water	Brisbane South
68	17	3	275134359rep	M. abscessus	Cutaneous/soft t	Wide Bay
69	17	3	341211044rep	M. abscessus	Cutaneous/soft t	South Coast
70	17	3	328444147rep	M. abscessus	Surgical wound	Brisbane South
71	17	3	336395997rep	M. abscessus	Pulmonary CF	Brisbane East (B
72	18		318540128	M. abscessus	Pulmonary	Brisbane North
73	19		328447600rep	M. abscessus	Cutaneous/Soft t	Brisbane North
74	20	4	387640523rep	M.a bscessus	Surgical wound	Brisbane Central
75	20	4	297683281	M. abscessus	Pulmonary	Brisbane West
76	20	4	SP110 R	M. abscessus	Municipal water	Brisbane West
77	20	4	366921511	M. abscessus	Pulmonary	Brisbane East
78	21	4	379714112rep	M. abscessus	Pulmonary	Sunshine Coast

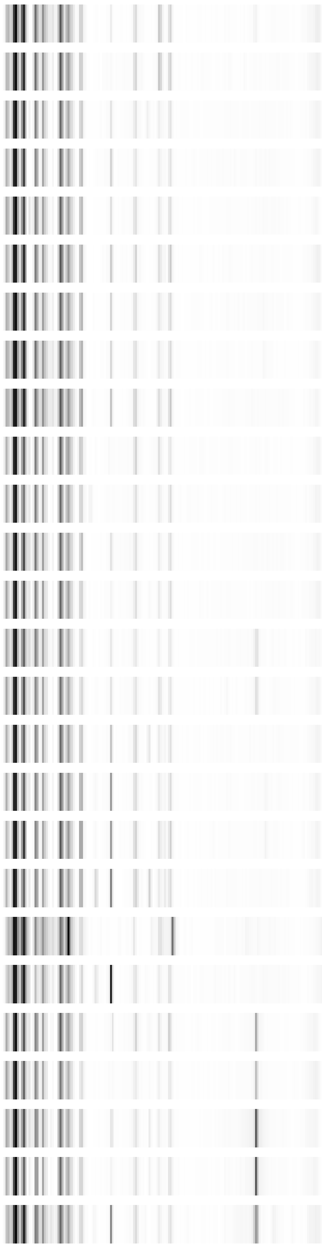

Similarity Line: 96.7%
◆ Discordant based on Sim Line: 2

Diversilab v3.4
PC
Analysis Report #696

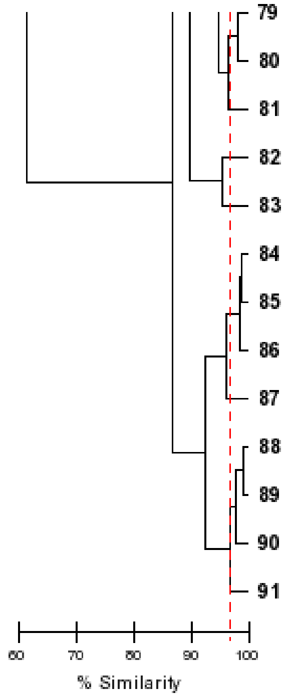

#	P	G	Sample ID	Species	Source	Location
79	22	4	SP114 WCPC R	M. abscessus	Municipal water	Brisbane South
80	22	4	280885764rep	M. abscessus	Pulmonary	Brisbane West
81	23	4	275138510	M. abscessus	Cutaneous/Soft t	South Coast
82	24	5	274804151	M. abscessus	Surgical wound	Brisbane Central
83	25	5	274802998	M. abscessus	Cutaneous/Soft t	South Coast
84	26	6	275136552	M. abscessus	Pulmonary	Brisbane North
85	26	6	29387102	M. abscessus	Pulmonary	Brisbane West
86	26	6	275133697rep	M. abscessus	Pulmonary	Brisbane South
87	27	6	328447776rep	M. abscessus	Pulmonary	Brisbane South
88	28		390900825rep	M. abscessus	Pulmonary	Brisbane South
89	28		328447798rep	M. abscessus	Pulmonary	Brisbane South
90	28		427760051rep	M. abscessus	Pulmonary	Brisbane North
91	28		275134478	M. abscessus	Pulmonary	Brisbane North

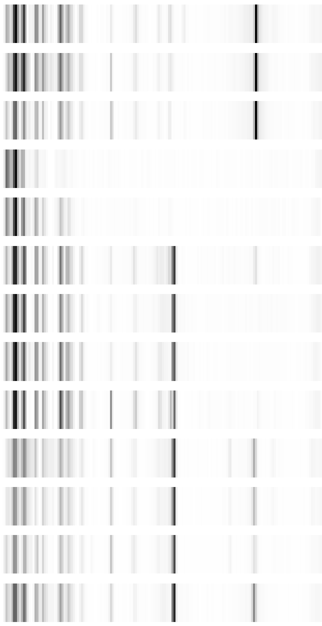

Similarity Line: 96.7%
◆ Discordant based on Sim Line: 2

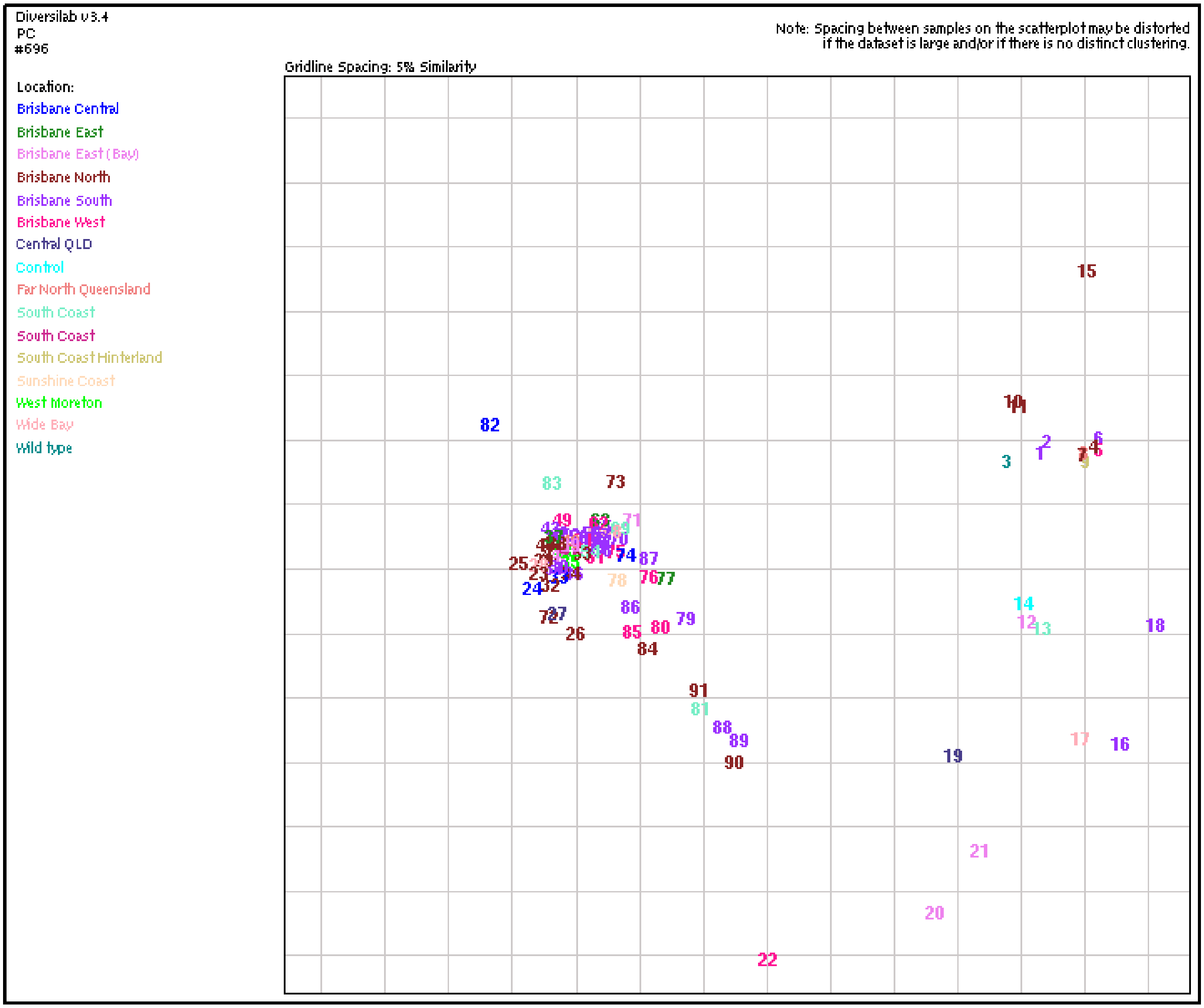
Diversilab v3.4
PC
#696
Note: Spacing between samples on the scatterplot may be distorted if the dataset is large and/or if there is no distinct clustering.
Gridline Spacing: 5% Similarity
Location:
Brisbane Central
Brisbane East
Brisbane East (Bay)
Brisbane North
Brisbane South
Brisbane West
Central QLD
Control
Far North Queensland
South Coast
South Coast
South Coast Hinterland
Sunshine Coast
West Moreton
Wide Bay
Wild type
